# Dysregulated MicroRNAs in the Pathogenesis of Systemic Lupus Erythematosus: A Comprehensive Review

**DOI:** 10.7150/ijbs.74315

**Published:** 2023-05-08

**Authors:** Daeun Choi, Jimin Kim, Jae Won Yang, Ji Hong Kim, Seoyeon Park, Jae Il Shin

**Affiliations:** 1 Yonsei University College of Medicine, Seoul, Republic of Korea.; 2 Department of Nephrology, Yonsei University Wonju College of Medicine, Wonju, Republic of Korea.; 3 Department of Pediatrics, Yonsei University College of Medicine, Seoul, Republic of Korea.

**Keywords:** microRNA (miRNA), systemic lupus erythematosus (SLE), T lymphocyte, B lymphocyte, plasmacytoid dendritic cell (pDC)

## Abstract

Systemic lupus erythematosus is a chronic autoimmune disease of which clinical presentation is vastly heterogeneous, ranging from mild skin rashes to severe renal diseases. Treatment goal of this illness is to minimize disease activity and prevent further organ damage. In recent years, much research has been done on the epigenetic aspects of SLE pathogenesis, for among the various factors known to contribute to the pathogenic process, epigenetic factors, especially microRNAs, bear the most therapeutic potential that can be altered unlike congenital genetic factors. This article reviews and updates what has been discovered so far about the pathogenesis of lupus, while focusing on the dysregulation of microRNAs in lupus patients in comparison to healthy controls along with the potentially pathogenic roles of the microRNAs commonly reported to be either upregulated or downregulated. Furthermore, this review includes microRNAs of which results are controversial, suggesting possible explanations for such discrepancies and directions for future research. Moreover, we aimed to emphasize the point that had been overlooked so far in studies regarding microRNA expression levels; that is, which specimen was used to assess the dysregulation of microRNAs. To our surprise, a vast number of studies have not considered this factor and have analyzed the potential role of microRNAs in general. Despite extensive investigations done on microRNA levels, their significance and potential role remain a mystery, which calls for further studies on this particular subject in regard of which specimen is used for assessment.

## Introduction

Systemic lupus erythematosus (SLE) is a chronic autoimmune disease predominantly affecting the female sex, starting frequently at reproductive age. The course of disease in most cases involves alternating periods of remission and relapse, resulting in a life-long inflammatory condition 1. Often referred to as the prototype of systemic autoimmune diseases, SLE is characterized by elevated levels of circulating autoantibodies and involvement of multiple organs, including the skin, joints, blood vessels, lungs, heart, brain, and kidneys 2. Clinical presentations are heterogeneous, ranging from mild skin rashes to life-threatening renal failures. Etiology is thought to be multifactorial and is associated with genetic predispositions and environmental triggers, including UV light, infection, smoking, and drugs 3.

Contribution of both genetic and environmental factors to the initiation of SLE has been continuously described in the literature. However, as the nonspecific environmental factors are commonly exposed to the general population and yet, only a small percentage of people present with symptoms of lupus 4, genetic contributions to the disease have been investigated extensively, especially the field of epigenetic modifications. Epigenetic modification is defined as changes made in the chromosome without altering DNA sequence, which allows the biological system to affect gene expression levels in response to environmental signals and eventually result in a stably heritable phenotype 5. Increasing studies of epigenetics have shed light on a new perspective to describe the insufficiently-understood mechanisms of genetic and environmental factors, as seen in the low penetrance of lupus in monozygotic twins (25-45%) 6.

Means of epigenetic alterations are frequently depicted by three principal categories: DNA methylations, histone modifications, and non-coding RNAs. Particularly, microRNAs (miRNAs), a typical example of a non-coding RNA, have increasingly gained awareness of its capability to control the other two principal means of epigenetic alterations, along with its direct implication in the pathogenesis of autoimmune diseases 7, 8. Moreover, unlike the irreversible character of gene mutations or chromosomal anomalies, the reversibility of epigenetic mechanisms holds therapeutic potential, which is another aspect why this particular field bears much significance 9.

In this review, we summarize the expression levels of dysregulated miRNAs in SLE patients with or without renal complications, taking into account the specific source of miRNA samples. We aimed to focus on the implication of these miRNAs in the pathogenesis of SLE.

## MicroRNA biogenesis and mechanism of action

MicroRNAs (miRNAs) are a class of small (~22 nucleotides), non-coding regulatory RNAs that are highly conserved among different species and has a crucial role in regulating the expression of its target genes at a post-transcriptional level. According to miRNA target gene studies, a single miRNA may have multiple target genes and vice versa, thus contributing to the complex yet fine-tuned regulatory network 10.

The biogenesis pathway of miRNAs involves a stepwise cleavage of long primary transcripts into smaller RNAs. In the canonical pathway, primary transcripts of miRNA coding genes (pri-miRNAs; pri-mir) are typically generated by RNA polymerase II 11. Pri-miRNAs contain hairpin-like secondary structures, which are cleaved off and processed into ~70-nucleotide (nt) precursor miRNAs (pre-miRNAs; pre-mir) by the nuclear microprocessor complex, comprised of the nuclear RNase III enzyme Drosha and its dsRNA-binding cofactor DGCR8 (DiGeorge syndrome critical region gene 8) 12. Some miRNAs are produced through non-canonical pathways in which hairpin structures are cleaved to produce pre-miRNAs via spliceosome-dependent mechanisms, bypassing the microprocessor machinery.

After transportation to the cytoplasm via exportin-5 (XPO5), pre-miRNA hairpins are further cleaved by the cytoplasmic RNase III Dicer and its dsRNA-binding cofactor TRBP (TAR-RNA binding protein), consequently yielding ~22-nt double-stranded RNAs 13, 14. This dsRNA molecule was conventionally termed as the miRNA/miRNA* duplex, for the more abundant strand was known to function as a guide strand for target gene silencing (miRNA), while the other strand was known to be degraded and was considered merely as a passenger strand (miRNA*). However, recent studies have provided evidence for the abundance and potential regulatory role of some miRNA* sequences 15. This led to the application of the current miRNA nomenclature, miRNA-5p and -3p, each corresponding to the 5'- and 3'- arm of the pre-miRNA hairpin.

**Figure 1:**
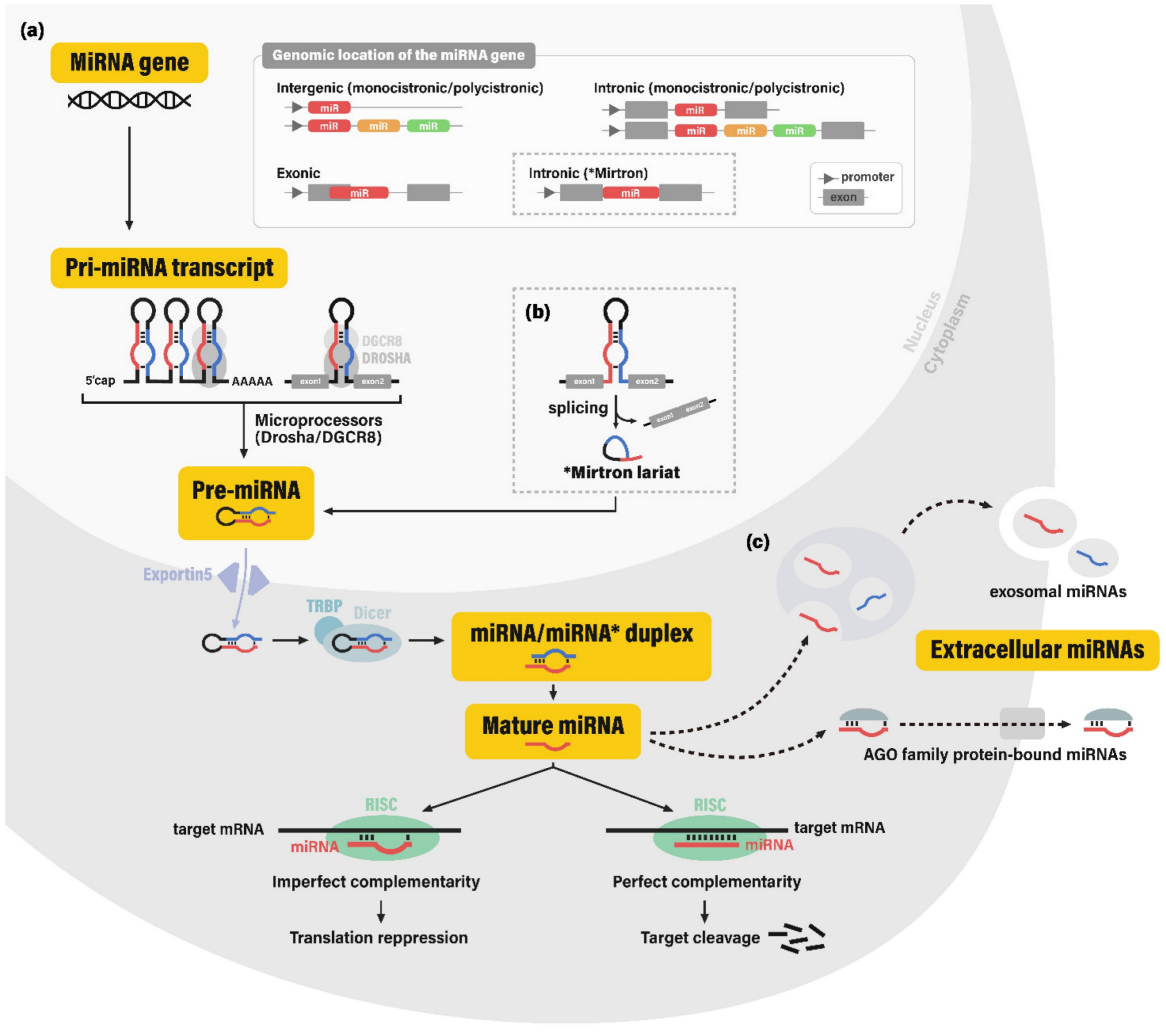
** Biogenesis of microRNAs.** (a) MicroRNA (miRNA) genes are reported to be encoded in intergenic, intronic, and exonic regions. In the canonical pathway of miRNA biogenesis, these miRNA genes are transcribed into primary microRNA transcripts (pri-miRNAs), which are cleaved by the microprocessor complex (nuclear RNAase III enzyme Drosha and its cofactor DGCR8) into hairpin-shaped RNA molecule pre-miRNA. After transportion to the cytoplasm via nuclear membrane portein exportin-5, pre-miRNA is cleaved by the cytoplasmic RNase III Dicer and its cofactor TRBP into the miRNA/miRNA* duplex, or more recently termed as miRNA-5p and miRNA-3p RNA strands. The resulting single-stranded mature miRNA is incorporated into the RNA-induced silencing complex (RISC) and complementarily binds to its target gene. Depending on the level of complementarity, binding of RISC leads to the translation repression or cleavage of the target gene. (b) Some miRNAs are produced bypassing the microprocessor machinery, among which the most well-known process involves the mirtron molecule; hairpin-shaped RNA molecules originating from the intronic regions spliced from mRNAs through spliceosome-dependent mechanisms. (c) It is still controversial whether extracellular miRNAs are merely byproducts resulting from cell death or molecules secreted through elaborate regulatory systems. Nonetheless, miRNAs in extracellular fluids are generally observed in two forms: exosomal miRNAs and AGO (Argonaute) protein bound miRNAs. Abbreviations: miRNA, microRNA; DGCR8, DiGeorge syndrome critical region gene 8; TRBP, TAR-RNA binding protein.

For exertion of its regulatory function, the single-stranded mature miRNA is finally incorporated into the RNA-induced silencing complex (RISC), which contains a member of the endonuclease Argonaute family (AGO. The mature miRNA strand plays a guiding role in the RISC, by recognizing their mRNA target sequences through complementary base pairing. RISC negatively regulates its target mRNA, via destabilization of the mRNA molecule, along with inhibition of the translation initiation process 16, 17 (**Figure 1**).

## Pathogenesis of SLE

### Overview

SLE is a multifactorial disease, in which both genetic and environmental factors are believed to contribute to disease progression. Epidemiologic studies have shown that environmental factors, such as ultraviolet (UV) light, cigarette smoking, and infection are associated with the development of lupus. These environmental risk factors are substances commonly found in everyday life but only a small portion of people who are exposed to these triggers present with symptoms of SLE, suggesting that people who develop SLE symptoms bear a predisposing factor, namely, genetic susceptibility. Indeed, there seems to be a strong link between genetics and the development of SLE, as demonstrated in the studies of different ancestries 18-22, siblings, and monozygotic/dizygotic twins. The complex interplay between genetic and environmental factors has not been fully elucidated, thus, we hereby present a brief summary of the pathogenesis of SLE about what has been discovered so far.

There are numerous aspects in the immune system which may contribute to loss of self-tolerance as the human body's immune reaction is very much complex and fine-tuned even to the slightest difference. Nevertheless, the fundamental phenomenon that underlies all hypotheses remains untouched: loss of tolerance to self-antigens and production of self-antigen-specific autoantibodies 23. The immune system becomes sensitized to self-antigens, such as nuclear proteins, cytoplasmic proteins, and membrane components of the body's own cell. Autoantibodies detected in SLE patients most commonly target antigens of nuclear origin, such as double-stranded DNA (dsDNA), histone proteins, Smith antigen (Sm), snRNP, and Sjögren's-syndrome-related antigen A and B (Ro/SS-A, and La/SS-B, respectively) 24, 25. These antinuclear antibodies (ANAs) are considered a serologic hallmark of SLE and are utilized as means of prognostic assessment of the disease 25, 26.

The initial contributing factor of this phenomenon of sensitization to self-antigens is an abnormal increase of exposure of self-antigens to the immune system, which would not have occurred as much in the absence of a pathologic condition. This feature is attributed to dysregulation of cell deaths, such as the classical apoptosis and neutrophil-specific death called NETosis, along with defective clearance of the remaining debris 27. With increased cell death and impaired clearance in SLE patients, the imbalance leads to accumulation of extracellular self-antigens, a state of chronic inflammation, and ultimately, stochastically generated autoreactive immune cells.

**Figure 2:**
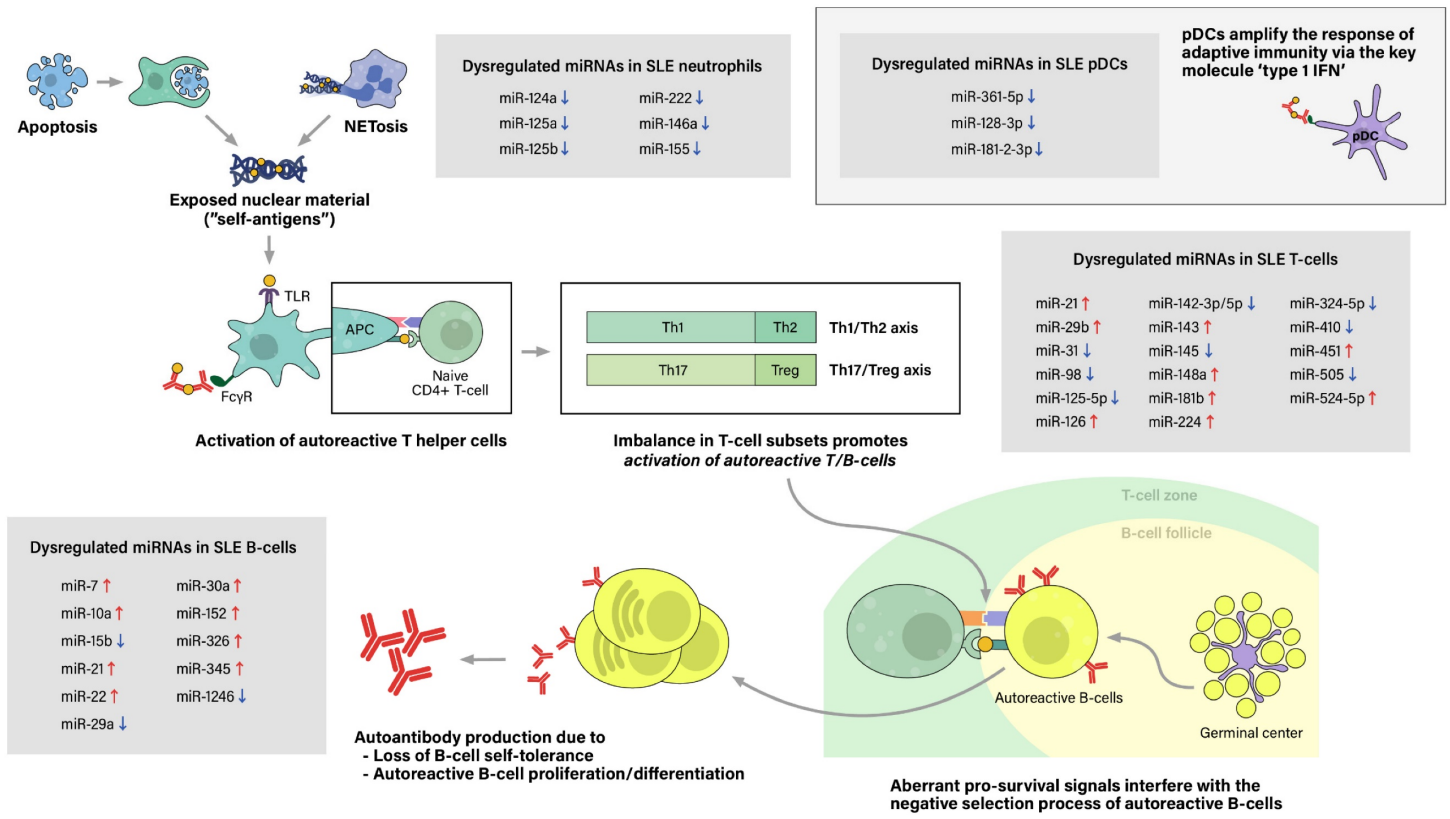
** Pathogenesis of Systemic lupus erythematosus (SLE) and associated miRNAs upregulations/downregulations in SLE immune cells.** Increased cell death via the classical apoptosis and the more recently investigated NETosis, along with defective clearance of the debris, lead to increased exposure of self-antigens to the immune system, and consequently, activation of autoreactive immune cells. SLE immune cells have been reported to show signs of hyperactivation, including increased numbers, increased sensitivity to stimulatory signals, and dysregulated secretion of chemokines/cytokines. Furthermore, altered ratio of T helper cell subtypes skew T-cell response towards B-cell activation. Moreover, type 1 interferon (IFN), a key immunoregulator mainly produced by plasmacytoid dendritic cell (pDCs), have also been observed to be upregulated in SLE patients. As a result, aberrant levels of autoantibodies and chemokine/cytokines lead to chronic inflammation, tissue damage, and end-organ injuries, clinically presenting as the well-known autoimmune disease.

The process of sensitization begins as immature antigen presenting cells (APCs), such as immature macrophages or conventional dendritic cells (cDCs), recognize, engulf, and display self-antigens on their surfaces. The antigens are presented to naive T-helper (Th) cells in nearby lymph nodes, resulting in activation of the autoreactive Th-cell population into different types of Th cells. Amongst them, activated Th2 cells stimulate autoreactive B-cells, thus resulting in proliferation of plasma cells that produce pathogenic autoantibodies. In SLE patients, both T-cells and B-cells have been reported to show signs of hyperactivation: increased numbers of activated cells found in the peripheral blood, increased sensitivity to stimulatory signals, and skewing of T-cell response towards B-cell activation 28. Moreover, aberrant levels of cytokines, such as high levels of type 1 interferon, also promote T/B-cell activation and humoral immunity. Plasmacytoid dendritic cells are known to take part in this particular feature (**Figure 2**).

### Step 1. Excessive exposure of self antigens to the immune system

Accumulation of extracellular self-antigens in SLE is attributable to increased cell deaths and defective clearance of their remnants, in which associated cell deaths include classical apoptosis and neutrophil-specific NETosis. The classical apoptosis process is triggered via the death-receptor-mediated (extrinsic) pathway or the mitochondrial-oxidative-stress-dependent (intrinsic) pathway, both pathways ultimately activating caspases and resulting in apoptotic blebbing of the cell 29. Apoptotic cells are recognized by phagocytic cells, such as macrophages, then are immediately engulfed and degraded to prevent the exposure of potential self-antigens to the immune system. Thus, in healthy individuals, apoptosis takes place in an immunologically silent manner 30. However, *in vivo* and* in vitro* studies have reported impairment of phagocytic functions in macrophages, monocytes, and neutrophils collected from SLE patients. Consequently, the uncleared apoptotic blebs lose their cellular integrity and undergo a necrotic phase called secondary necrosis, resulting in exposure of intracellular proteins to the immune system 31.

Another type of dysregulated cell death in SLE is NETosis. Traditionally, cell death of neutrophils has been referred to either programmed cell death (apoptosis) or non-programmed cell death (necrosis). Adding to this, another mean of cell death has been brought to light recently, called NETosis. NETosis is the process of neutrophils forming neutrophil extracellular traps (NETs), activated to trap and kill microbes when the pathogen outsizes the phagocytosis-possible capacity 32, 33. The neutrophil extracellular trap (NET) comprises decondensed chromatin fibers with histone proteins, cytoplasmic proteins, and contents of neutrophil granules. After NETosis, NETs are partially degraded by extracellular DNase 1, and these preprocessed NETs are recognized and cleared by macrophages with the assistance of C1q opsonization 34. While NET degradation is initiated promptly in healthy individuals, enabling a immunologically silent process, increased NETosis and inefficient clearance of its remnants observed in SLE patients result in increased exposure of NET components to the immune system.

Despite growing interest in NETosis, there is limited data on the epigenetic aspect of lupus neutrophils. Pérez-Sánchez et al. reported that miR-124a, 125a, 222, 125b, 146a, 155 are downregulated in neutrophils from SLE patients compared to neutrophils from healthy controls 35. However, the linkage between microRNA dysregulation and abnormal neutrophil function, particularly NETosis, has not been assessed, which calls for additional research to fill this gap.

### Step 2. Exaggerated immune response to self antigens

In lupus patients, adding to excessive exposure of self-antigens to the immune system, T lymphocytes and B lymphocytes, both components of adaptive immunity, have been reported to show signs of hyperactivation. Aberrant T-cell signaling and imbalance in T-cell subsets lead to unrestricted hyperactivation of B-cells, along with the impaired development of naive B-cells in germinal centers 36. These aberrant activities of adaptive immunity lead to generation of excessive self-reactive immune cells, autoantibodies, inflammations, and consequently, end-organ damage of the disease.

#### Dysregulated miRNAs in T-cells and their potentially pathogenic roles

##### Overview of the mechanism of T-cell development

The role of CD8+ T-cells in the pathogenesis of SLE is not well-known 37. However, hyperactivation and increased levels of CD4+ T 'helper' cells have been observed in the peripheral blood of SLE patients. T helper cells are key regulators of the adaptive immune system. Not only do they help B-cells gain function to produce antibodies, but also shape the general landscape of the body's immune response to presented antigens. As a naïve CD4+ T-cell circulates within the lymph-vascular system, it encounters its cognate antigen displayed by antigen-presenting cells (APCs), especially dendritic cells (DCs) that have migrated from different tissues of the body. This interaction usually takes place in secondary lymphoid organs, such as the spleen, lymph nodes, and mucosal associated lymphoid tissues, consequently leading to alterations in the T-cell signaling pathway 38. A T-cell receptor (TCR) assembled with CD3 proteins (CD3δ, ε, γ, and ζ), recognizes its cognate antigenic peptide, and with the help of a secondary signal, initiates the intracellular signaling pathway in naive T-cells. Among the number of secondary signals that have been identified, CD28 on the T-cell binding to B7.1 (CD80) or B7.2 (CD86) on the APC is the key signal that specifically triggers proliferation of T-cells recognizing this particular antigen. Further differentiations into distinct lineages of effector CD4+ T-cells is determined by cytokine signaling.

##### Structural differences observed in lupus T-cells

Phosphorylation of immunoreceptor tyrosine-based activation motifs (ITAMs) in the CD3ζ chain is one of the chief features of the T-cell activation process. Lck, a T-lymphocyte-specific tyrosine kinase, targets tyrosine residues of ITAMs in CD3ζ chains. These phosphorylated ITAMs act as binding sites for the 70-kDa zeta chain-associated protein (ZAP-70), another type of tyrosine kinase, and this CD3ζ-ZAP-70 interaction further leads to intracellular signaling cascades resulting in calcium influx into T-cells 39, 40. Thereafter, nuclear factor of activated T-cells (NFAT), a family of transcription factors known to be Ca-dependent, becomes dephosphorylated and activated via the Ca2+ mediated calmodulin-calcineurin pathway. NFAT then translocate into the nucleus, binds to the promoter of several genes and initiates their transcription, therefore inducing the production of cytokines essential for the proliferation and expansion of T-cells, such as IL-2, and promoting the expression of co-stimulatory molecules on the surface of T cells, such as CD40L. In lupus T-cells, however, expression of CD3ζ chains is significantly decreased and are replaced by the γ chain of high affinity IgE receptor (FcRγ), which is structurally homologous with the CD3ζ chain 41, 42. Thus, in lupus T-cells, the FcRγ chain binds with another type of tyrosine kinase Syk instead of ZAP-70. Signaling between FcRγ and Syk is known to be much stronger than it between CD3ζ and ZAP-70, causing hyperactivation of TCR signaling, thus resulting in increase of Ca2+ influx into SLE T-cells and elevation of NFAT level in the nucleus 41. However, consequences due to increased expression of NFAT are not as predictable; due to the overexpression of NFAT and its binding to its target promoters, CD40L gene is more easily activated, whereas production of IL-2 is observed to be lower in lupus T-cells. Several explanations have been suggested on this unexpected downregulation of IL-2 in lupus T-cells. First, since CD40L-CD40 signaling induces the differentiation of naive T-cells into Th17, it may contribute to the imbalance of Th cell subsets in SLE and dysregulation of cytokine levels. Second, decrease in IL-2 levels may be due to the increase of phosphorylated cAMP-responsive element modulator (p-CREM) due to Ca2+ /calmodulin-dependent kinase IV, which binds to the promoter of IL-2 gene and inhibits the production of IL-2 43.

Another known feature of lupus T-cells involves their surface morphology during activation and entry into targeted inflamed tissues. Immune cells, specifically, leukocytes and lymphocytes, have a distinct function being able to quickly undergo multiple steps of polarization on activating cues, adhesion to endothelial tissues, and migration into sites of inflammation. Polarization of a lymphocyte refers to the morphological transition of a freely circulating cell to an endothelium-adhering cell with a specialized surface site called uropods. Various adhesion molecules, such as CD44, are concentrated in uropods, enabling them to stabilize migrating lymphocytes. The polarization and uropod formation process are known to be mainly regulated by ezrin, radixin, and moesin (ERM) proteins; serine/threonine kinases, such as Rho-associated protein kinase (ROCK) or protein kinase C (PKC) phosphorylates threonine residues of ERM proteins, then, phosphorylated ERM proteins set off a signaling cascade leading to alterations of cytoskeletal assembly 44, 45. In lupus T-cells, ERM proteins show increased levels of phosphorylation along with overexpression of surface molecule CD44 compared to T-cells of healthy controls, promoting T-cell adhesion and TCR-CD3 signaling in lupus patients 45. Furthermore, according to recent findings, ROCK has been found not only to regulate cytoskeletal assembly of lymphocytes, but also the effector function of CD4+ T-cells. Biswas et al reports that ROCK2, an isoform of Rho kinase, was activated in murine T-cells in Th17-cell dominant environments and phosphorylated interferon regulatory factor 4 (IRF4), a transcription factor involved in IL-17 and IL-21 gene expression. ROCK2-mediated activation of IRF4 leaded to IL-17 and IL-21 production, which are both key pathogenic cytokines known to contribute to autoimmune disorders, and Th17 cell differentiation from naïve T-cells.

Lastly, enhanced activity of phosphoinositide-3 kinase (PI3K), a key component in the PI3K/Akt/mTOR pathway, was observed in T cells of SLE patients. In the well-known PI3K/Akt/mTOR pathway, PI3K phosphorylates PIP2 (phosphatidylinositol 4,5-bisphosphate), then, phosphoinositide-dependent protein kinase 1 (PDK1) binds to the phosphorylated product, PIP3, and activates Akt, also known as protein kinase B (PKB). Akt, as the main molecule in the PI3K signaling pathway, has various downstream effects, amongst mammalian target of rapamycin (mTOR) is especially the molecule of interest in the field of autoimmunity. Phosphorylated Akt activates mTOR, which has been widely investigated for its role in naïve T-cell activation, differentiation into Th1, Th2, Th17 cell lineages, and inhibition of regulatory T-cells and T-cell anergy. Studies have shown that Akt levels are increased in T cells and B cells of SLE patients and that there lies a positive correlation between Akt/mTOR activation in B cells and disease severity 46, 47.

##### T-cell subset alterations observed in lupus patients

Upon stimulation by certain cytokines and membrane-bound molecules of APCs, the naïve T-cell undergoes clonal expansion and differentiation into functionally distinct CD4+ effector T-cell lineages 38. Different types of helper T-cells include the 'classical' effector T helper 1 cells (Th1 cells), T helper 2 cells (Th2 cells), T helper 17 cells (Th17 cells), the immunosuppressive regulatory T-cells (Treg cells), and lastly, T follicular helper cells (Tfh cells). The subsets can also be defined based on their cytokine secretion profiles: Th1 cells chiefly produce IFN-γ, IL-2, and tumor necrosis factor β (TNF-β), whereas Th2 cells produce IL-4, IL-5, IL-6, IL-10 and IL-13 48. It has long been established that Th1 cells activate classical macrophages and amplify the body's immune response against intracellular pathogens, such as viruses, while Th2 cells stimulate mast cells and eosinophils, promote antibody production, and protect the body against extracellular pathogens, such as multicellular parasites. While the Th1/Th2 balance constitutes one axis of the immune homeostasis, another more recently recognized axis is the Th17/Treg balance. Th17 cells are characterized by the master transcription factor RAR-related organ receptor γ (RORγt) and are known to mainly produce IL-17, IL-21, IL-22, and IL-23, and plus, TNF-α and IL-6 when exposed to certain microenvironments 49, 50. It has been understood that Th17 cells play a major role in autoimmunity with their ability to recruit neutrophils and promote inflammatory responses. In contrast, Treg cells produce anti-inflammatory cytokines, such as IL-10 and transforming growth factor β (TGF-β), inhibiting a variety of immune cells and consequently inhibiting autoimmunity. IL-17, a key effector cytokine of Th17, are also known to promote B-cell differentiation and survival via upregulation of the B lymphocyte stimulator (BLyS) 36.

Imbalance in T-helper-cell subsets (Th1/Th2/Th17/Treg) has been suggested to contribute to the pathogenesis of SLE. However, it is still controversial which T-helper-cell subset and related cytokines mainly contribute to the pathogenic process 51. Regarding the Th1/Th2 balance axis, the traditional understanding was that function of Th2 cells are dominant in SLE patients compared to Th1 cells, promoting inappropriate development of autoantibodies in B-cells. However, recent studies report that Th1 cells may be involved in the pathogenic process as well, as exacerbation of lupus was observed after injection of IFN-γ in a lupus mouse model. Dolff et al has also reported the dominance of Th1 cells in the chronic stages of SLE, especially in patients with lupus nephritis IV 52. Meanwhile, regarding the more recently recognized Th17/Treg balance axis, it has been reported that T-helper-cells in lupus patients show skewing towards Th17 cells, as distinct cytokine profiles were observed: decreased IFN-γ, TGF-β and increased IL-17, each distinct cytokines of Th1 cells, Treg cells, and Th17 cells, respectively 51.

Expanded populations of Th17 cells have been reported in SLE patients, and the pathogenic role of these cells are well indicated in several studies. Increased levels of IL-17 and Th17 cells were found in the skin lesion biopsies of SLE patients and urinary sediment and kidney biopsies from patients with lupus nephritis. Treg cells, known to suppress and modulate the activity of other T-cells, have also been reported to play a key role in the pathogenesis of SLE as impaired function of Treg cells can result in generation of autoantibodies and loss of immune tolerance. Lower ratios of Treg/Th17 were observed in active SLE patients compared to healthy controls, and the deficiency of Treg cells were found to be correlated with SLE disease activity 49. Decreased levels and impaired function of Th17 cells in lupus patients have also been reported.

##### Dysregulated miRNAs and their potential contributions to aberrant T-cell activity

In regard of the aforementioned roles of T-cells in SLE pathogenesis, we ought to identify how dysregulated miRNAs in T-cells contribute to T-cell hyperactivation and the onset of the disease. According to Lu M et al, downregulation of miR-145 in lupus T-cells suppressed API5 expression that lead to T-cell activation-induced cell death, and upregulation of miR-224 enhanced expression of STAT-1 in SLE T-cells, causing lupus nephritis 53. miR-524-5p was also significantly overexpressed in SLE T-cells, increasing the production of IFN-γ in activated T-cells. It also diminished protein expression of Jagged-1 and Hes1 in T-cells, which are both related to Notch signaling pathway, resulting in increased SLE disease activity 54. Expression level of miR‐31 was decreased in SLE T-cells, leading to reduced expression of IL‐2 by suppressing NFAT and IL-2 promoter activity. RhoA expression was negatively correlated with miR-31 level, and therefore was upregulated in SLE T-cells 55. miR-410, which significantly suppressed the expression of IL-10 by targeting STAT3 activity, was decreased in SLE T cells. Furthermore, the level of miR-410 was also found lower in CD4+ T cells 56.

Pan et al reported that miR-21 and miR-148a were both overexpressed in CD4+ T cells. They were found to suppress DNMT1 by targeting RASGRP1 and its protein coding region respectively, promoting demethylation of CD4+ T cells and resulting in enhanced expression of methylation-sensitive autoimmune-associated genes such as CD70 and LFA-1 57. Previous study has also shown that inhibition of BDH2, a direct target of miR-21-5p, contributes to DNA hypomethylation in CD4+ T cells by increasing intracellular iron concentration 58. It has been reported that PDCD4, another target of miR-21, might also play a central role in SLE 59. Up-regulation of miR-29b and miR-126 in CD4+ T cells also appeared to play an important role in SLE pathogenesis by suppressing DNMT1 and inducing hypomethylation, thereby enhancing expression of particular genes such as CD70 and CD11a that contributes to the autoreactive status of T cells 60, 61. miR-125a(-5p), which negatively regulates several effector T-cell factors including STAT3, IFN-γ and IL13, was downregulated in peripheral CD4+ T cells in SLE, leading to destabilization of Treg mediated immune homeostasis 62. miR‐142‐3p and miR‐142‐5p in CD4+ T cells of SLE patients were significantly downregulated due to DNA methylation of its promoter region, increasing translation level of its target proteins, SAP, CD84, and IL-10, and thereby causing overactivation of T cells and B cells 61, 63. Downregulation of miR-98 was found to contribute to SLE pathogenesis by inducing Fas-mediated apoptosis of CD4+ T cells 64. According to R Martinez-Ramos, levels of miR-224 and miR-143 were significantly overexpressed in CD4+ T cells of inactive, asymptomatic SLE patients. He suggested that overexpression of miR-224 could have induced IRF4 overexpression and increased secretion level of IL-21, causing hyper responsiveness of IL-21 dependent B cells 65. However, no correlation of target genes involved in autoimmune response with miR-143 has been found yet 65. miR-633 level was downregulated in SLE CD4+ T cells, contributing to SLE pathogenesis by targeting AKT1 and activating AKT1/mTOR pathway, which is known to play an important role in the activation of T cells 66. Additionally, Ming Zhao et al have shown that downregulation of miR-142-3p, miR-505, miR-324-5p, and upregulation of miR-126, miR-451, miR-181b were also observed in T cells of SLE patients regardless of their clinical phenotypes including skin lesions, chronic renal pathology, and polyarticular disease 67.

Number and function of Treg cells are known to be impaired in SLE and other autoimmune diseases 68. Sun et al reported that miR-326 expression level was significantly upregulated in Treg cells of new-onset SLE patients, positively correlated with CRP and anti-C1q antibody, and negatively correlated with Ets 69.

#### Dysregulated miRNAs in B-cells and their potentially pathogenic roles

##### Overview of the mechanism of B-cell development

Autoantibody production due to loss of B-cell self-tolerance and the alteration of B-cell differentiation is one of the major immunological aberrations in SLE pathogenesis. In lupus patients, B-cells are hyperactivated, increased in the peripheral blood, and are more prone to polyclonal activation by its specific antigens, resulting in the formation of a wide range of autoantibodies 3. B-cells are lymphocytes capable of secreting immunoglobulins, or antibodies, that specifically recognize proteins of potentially invasive pathogens, also called antigens. An immunoglobulin comprises two heavy chains and two light chains, both of which undergo gene rearrangements in the developmental stages, consequently generating an enormously diverse antibody repertoire in a single human being. The B-cell development process can be divided into two phases: the antigen-independent phase within the bone marrow (stem cells, pro-B cells, pre-B cells, and immature B-cells) and the antigen-dependent phase within secondary lymphoid tissues (naive mature B-cells, activated B-cells) 70. The intermediate phase of immature B-cells between the two phases is referred to as transitional B-cells, identified as CD19^+^ CD24^high^ CD38^high^ lymphocytes 71. These transitional B-cells further evolve into CD19^+^ CD24^int^ CD38^int^ naive mature B-cells, which remain quiescent and circulate through the peripheral blood and lymphatic system until they meet their specific antigens in secondary lymphoid organs (SLOs), such as lymph nodes, the spleen, and gut-associated lymphoid tissue (GALT) 72. Thereafter, an antigen-dependent process of B-cell expansion and differentiation takes place in order to yield high-affinity antibody-producing plasma cells and memory cells 70.

Throughout the early developmental stages of B-cells, immunoglobulin gene recombination takes place, and amongst them, malfunctioning or self-reactive combinations are eliminated through multiple immunological checkpoints. The resulting combinations of immunoglobulin heavy chains and light chains are assembled and presented on the cell membrane of immature B-cells, and are called B-cell receptors (BCRs). For maturation and proliferation, a developing B-cell requires pro-survival signals, including B-cell activating factor (BAFF, also known as BLyS), tonic BCR signaling, and certain cytokines, such as IL-7 73. These signals synergize and promote the survival of B-cells at different stages of development. For example, while early developing B-cells (pro-B cells, pre-B cells) require signals through IL-7 receptors in order to survive, the impact of IL-7 on immature B-cells is minimal 74-76. Also, BAFF signaling is known to play its role starting from the transitional stage of B-cells, while in the survival of naive mature B-cells, it has been reported that both tonic BCR signaling and BAFF signaling are necessary. Of these three pro-survival signals, BAFF has been especially emphasized of its role in autoimmune diseases. Increased serum levels of BAFF and/or its homolog APRIL (a proliferation-inducing ligand) have been reported in lupus patients, some of which had also been found to be positively correlated with autoantibody titers 77, 78. Studies have discovered that excess BAFF promotes naive B-cell survival, interferes with the negative selection process of autoreactive B-cells within GCs, and facilitates immunoglobulin class switching, as a result, enhancing aberrant immune responses 79-82. There have also been promising results in murine studies regarding the therapeutic potential of BAFF/APRIL inhibitors in treating SLE, however, clinical trials of anti-BAFF antibodies have failed to show significant results so far 77, 78, 83. Nonetheless, BAFF/APRIL appears to play a key role in the pathogenesis of autoimmune diseases, including SLE, and many questions remain to be unsolved about its specific mechanism of action.

Adding to pro-survival signals, the fate of developing B-cells also depends on pathogen-associated molecules, especially in the antigen-dependent phase within SLOs. Naive mature B-cells express both IgM and IgD-BCR on their surfaces, and as they first encounter their cognate antigens presented by follicular dendritic cells (FDCs) in the B-cell follicles of SLOs, this leads to the internalization and presentation of the antigen on MHC class II molecules of B-cells. These antigen-presenting B-cells then migrate to the border of the T-cell zone and interact with CD4+ Th cells, which had already been primed by activated dendritic cells 70. Th cells further differentiate into T follicular helper cells (Tfh cells); a process driven by the upregulation of Tfh cell-associated transcription factors, including B-cell lymphoma 6 protein (BCL-6) 84. BCL-6 is a transcription factor that is crucial for the development of not only Tfh cells but also germinal center (GC) B-cells, playing a pivotal role in the germinal center reaction 85. Meanwhile, at the T-B border, stimulated B-cells differentiate into GC-independent memory B-cells, short-lived plasma cells, and GC B-cells, among which GC B-cells are the cells that participate in the GC reaction and generate high-affinity class-switched antibodies 70. Tfh cells, in combination with FDCs, play a key role in the formation of GCs, which are the very places where negative selection of autoreactive B-cells and positive selection of high-affinity B-cells take place, bearing the potential as culprit for self-reactive antibody generation in autoimmune diseases. According to multiple *in vivo* studies of lupus patients, increased levels of circulating Tfh cells were observed to be positively related with the disease activity and autoantibody titer of lupus 86, 87.

Although not fully understood, transitional B-cells seem to be closely related to IL-10-secreting regulatory B-cells (Breg cells), similarly secreting the anti-inflammatory cytokine IL-10. IL-10 suppresses the expansion and differentiation of Th1 and Th17 cells, thereby limiting production of pro-inflammatory cytokines 88-90. The general consensus is that transitional B-cells play a regulatory role in inflammatory conditions and prevent autoimmunity, as they are also known to take part in the differentiation of CD4+ FoxP3 Treg cells 88, 91. However, there have been some studies in which transitional B-cells were observed to secrete pro-inflammatory cytokines as well, for example, IL-6, which regulates the differentiation of Treg cells and promotes autoreactive responses of Th1 and Th17 cells 90, 92-95. Interestingly, despite the general understanding on transitional B-cells, studies on lupus patients have demonstrated elevated levels of CD19^+^ CD24^high^ CD38^high^ transitional B-cells 88, 92, 93, 96, 97 and decreased levels of the closely-related regulatory B-cells 98. In one of these studies, along with elevated levels of transitional B-cells, functional disabilities of these cells were also observed 88, providing a possible explanation for the conflicting results.

##### Dysregulated miRNAs and their potential contributions to aberrant B-cell activity

Regarding the previously mentioned roles of B-cells in the pathogenesis of SLE, we ought to identify how the dysregulated miRNAs in B-cells induce hyperactivation of B-cells and the onset of the disease. Increased expression of miR-7, miR-21, miR-22 in SLE B-cells were reported to play a critical role in B-cell hyper-responsiveness by down-regulating PTEN, an inhibitor of B cell signaling, and thereby enhancing BCR signaling. Moreover, IL-21, produced mainly by activated CD4+ T-cells, appeared to up-regulate miR-7 and miR-22 in both healthy and lupus B-cells, providing a positive feedback between activated T-cells and B-cells in lupus patients 99. However, IL-21 is also known to upregulate PTEN expression, making its relation between BCR signaling complex. A recent study has also revealed that the level of miR-30a in SLE B cells were significantly increased, negatively affecting the level of Lyn, a tyrosine kinase which negatively regulates BCR signaling by phosphorylating inhibitory receptors, which therefore leads to B-cell hyperactivation 100. MiRNA-152, which directly targets Kruppel-like factor 5 (KLF5) that binds to the promoter region of BAFF and inhibits its expression 101, was reported to be upregulated in B-cells of SLE patients, contributing to B-cell hyperactivation. Jin L et al. have shown that the upregulation of miR-326 also promotes B-cell hyperactivation by targeting Est-1, a negative regulator of B-cell differentiation 102. According to Martinez-Ramos R et al, miR-10a and miR-345 were overexpressed in B-cells from asymptomatic SLE patients. He expected that miR-10a has a possible inhibitory role on serum IL-8 level, which has been established to positively correlate with SLE disease activity, supporting the fact that SLE patients with high miR-10a in B-cells were asymptomatic and had low generation of autoantibodies from B-cells. Similarly, overexpressed miR-345 in inactive phase of SLE has a potential to inhibit IRF-8 in B-cells, a key transcription factor regulating B-cell differentiation 65.

MiR-15b, which directly targets Cyclin D3 (CCND3) that plays an important role in B-cell development and required for the transition of pro-B cell to pre-B cell, appeared to be downregulated in B cells. Ren D, et al. demonstrated that SLE patients showed significant upregulation of CCND3, due to the decrease of miR-15b in B-cells and the activation of TLR7, B-cell intrinsic receptor that senses single stranded RNA 103. Significant downregulation of miR-1246 was also reported in SLE B-cells compared to healthy controls. According to Luo et al, miR-1246 regulates the expression of Early B-cell factor 1(EBF1), which contributes to the proliferation of B-cells via the AKT signaling pathway. Therefore, decreased miR-1246 enhances EBF1 expression and B-cell activity. Also, activated B-cell's AKT-p53 pathway was reported to decrease the level of miR-1246, leading to further activation of B-cells 104. Level of miR-29a in lupus B-cells were downregulated compared to healthy controls. Et al. demonstrated that decreased miR-29a enhances the expression of its target gene, Crk-like protein (CRKL) in B-cells, resulting in increased secretion of IgG and activation of B-cells 105.

##### Role of plasmacytoid dendritic cells in systemic lupus erythematosus

Human dendritic cells are divided into two subsets: the 'classical' myeloid dendritic cells (mDC)s, and the plasmacytoid dendritic cells (pDCs) that are morphologically and functionally distinct from mDCs. On a molecular level, while mDCs express TLRs-1 to -6, -8 and -10 on their surface, pDCs have a less diverse profile of surface molecules, predominantly TLRs-7 and -9. Functionally, both dendritic cells engulf molecules of interest by endocytosis using their surface molecules, however, pDCs retain engulfed self-antigens for a longer time, enabling continuous production of type 1 IFN. Studies have shown that SLE patients have a normal or reduced frequency of mDCs compared to healthy controls. Studies regarding frequency of pDCs have not shown such consistent results, however recent research has shown that lupus pDCs exhibit more stimulatory activity and activated phenotypes 106. Although only recently recognized, the role of pDCs in the regulation of immune responses have been vigorously investigated throughout the years; while mDCs are professional antigen-presenting cells that links innate immunity to adaptive immunity, pDCs are “natural type 1 IFN-producers” that immune modulate and amplify the response of adaptive immunity via the key molecule 'type 1 IFN' 36, 106.

Formation and deposition of immune complexes containing autoantigens lead to endocytosis of pDCs and amplification of self-molecule directed immune responses. pDCs identify immune complexes by FcγRIIa, and the endosomal TLR7 and TLR9 plays a central role in the activation of pDCs, resulting in the production of type 1 IFN, a key cytokine of SLE pathogenesis 107. Upregulation of type 1 IFN arouses multiple effects that contribute to the activity of the disease, such as increased differentiation of precursor cells into mDCs, activation of Th cells, increased secretion of pro-inflammatory cytokines, upregulation of the B cell activating factor (BAFF), promotion of isotype switching in activated B-cells, and loss of immune regulatory function of Treg cells 36, 108. As a result, immune complexes lead to amplified inflammatory responses and complement activation, thereby injuring various systemic organs 23.

Despite the key role of type 1 IFN as master regulators in amplifying adaptive immunity and the relatively well accepted consensus that type 1 IFN levels are upregulated in lupus patients, not much research has been done on the epigenetic aspect of lupus pDCs. We have found one study that has investigated about dysregulation of miRNAs specifically in pDCs. It revealed that miR-361-5p, miR-128-3p, and miR-181-2-3p were significantly reduced in the pDCs of SLE patients, especially in those with high type 1 IFN levels 109. Moreover, the level of miR-361-5p was related with the disease activity (SLEDAI ≥4). Aberrations of miRNA expression in pDCs potentially may be able to explain the upregulated levels of type 1 IFN in SLE, which calls for additional investigations specifically on these master regulator immune cells.

## Extracellular microRNAs

Differential *in vivo* expression levels of miRNAs have been widely investigated since microRNAs were recognized as important regulators of disease pathogenesis in various fields, notably autoimmunity 110, 111. Traditionally, the intracellular concentration of miRNAs was the topic of concern. However, a mounting number of studies have expanded their focuses to extracellular distributions of miRNAs 112, 113. Extracellular miRNA concentrations are measured in easily obtainable clinical samples, including blood plasma, serum, and urine 114-117. According to current understandings, miRNAs are able to endure the nuclease-rich environment outside cells by complexing with AGO family proteins, or being packaged into extracellular vesicles, such as exosomes, microvesicles, and apoptotic bodies. These circulating miRNAs are partly regarded simply as byproducts after cell death, while some suggest their significance as means of cell-to-cell communication 118, 119. In the latter point of view, miRNAs are secreted in a regulated process and function as autocrine, paracrine, or even endocrine messengers to modulate cellular activities of their recipient cells 120. Although further research is needed, it is regarded that both hypotheses are feasible, since a majority of extracellular miRNAs could be non-specific byproducts while some selected miRNAs engage in cell-cell signaling 118.

Despite lack of understanding on the physiology or function, dysregulated levels of extracellular miRNAs in plasma, serum, urine, etc., has been vigorously investigated in recent years. Here we update and summarize the pathogenic potentials of the most commonly studied miRNAs: miR-21, miR-146a, miR-155, and miR-181a.

MiR-21, which is significantly overexpressed in CD4+ T-cells and is associated with DNA hypomethylation, was shown to be upregulated in the plasma of SLE patients 121. According to Chen X et al, circulating miRNA are mostly derived from circulating blood cells 115, and this result proposes that the increased level of circulating miR-21 might be due to selective secretion of upregulated intracellular miR-21 in CD4+ T-cells and the exposure of CD4+ T-cells to circulating plasma in inflammatory conditions. However, the specific mechanism of selective secretion remains unclear. Moreover, circulating miR-21 was also reported to be increased in patients with RA, indicating that it might not be specific for SLE 121. Plasma miR-21 level was highly correlated with SLE disease activity score, and therefore may be used as a biomarker to distinguish the severence of SLE 122, 123. Its level was also negatively correlated with serum C3,4 levels and IL-2, and positively correlated with IL-10 expression 122-124.

According to Wang et al, the serum and plasma levels of miR-146a in SLE patients were down regulated compared to healthy controls and negatively correlated with SLEDAI 121, 125. Furthermore, serum exosomal miR-146a have shown to be decreased in SLE patients, and previous studies have reported that serum exosomal miR-146a negatively regulates senescence of SLE bone marrow mesenchymal stem cells by suppressing the TRAF6/NF-KB signaling pathway 182, 183. These are consistent with the report that miR-146a, which is known to contribute to the disease by regulating the type 1 IFN pathway, is significantly decreased in the PBMC of SLE patients 126. Urinary extracellular miR-146a level was notably higher in SLE patients and was correlated with estimated GFR, but its correlation between clinical measurements remains unclear 125, 127. Hernandez have also reported that in SLE patients, urinary miR-146a existed primarily in the form contained in exosomes and were increased in patients with active lupus nephritis 128.

The serum and plasma level of miR-155 in SLE have also been reported to be decreased in SLE patients, but its urinary level was significantly higher in lupus specimens and highly associated with SLEDAI and proteinuria 127. Divekar has reported that miR-155 level was upregulated in T cells in lupus, which may seem contradictory to its low circulating level in plasma 129. However, since several studies have shown that apoptotic cells can transfer its microparticles and apoptotic bodies to other cell types, increased transfer of miR-155 might have resulted in miRNAs' reduced circulating level 130.

Whether the circulating level of miR-181a increases is controversial. Carlsen A. et al and Motawi T. have reported that the plasma level of miR-181a was significantly increased in SLE patients and observed a correlation between SLEDAI score 122, 131. miR-181a was found to mediate inflammatory factors such as IL-1β, IL-6 132, IL-8 133, and TNF-α 134 and play a critical role in regulating T cell and B cell differentiation and innate immunity response 135. According to Li H, serum level of miR-181a was also increased in SLE patients and was positively correlated with ESR, CRP, anti-dsDNA, complement C4 and SLEDAI levels 136. However, several studies have observed downregulation of miR-181a in SLE patients. Its expression was found to be decreased in peripheral blood of Egyptian pediatric SLE patients, which may indicate that patterns of disease expression may differ in childhood onset and adult SLE patients 122, 137. In addition, Zhang H. et al has also observed a significant decrease of plasma miR-181a in SLE patients compared to healthy controls 138 (**Table 1-3**).

## Concluding remarks and future perspectives

MiRNAs have been widely investigated in search for their roles in the pathogenesis of SLE. Many studies on dysregulation of miRNAs compare miRNA levels of lupus patients and healthy controls, then identify potentially pathogenic target genes using multiple databases that predict biological targets and functions of miRNAs (ex. TargetScan, miRDB). The most commonly studied miRNAs, also regarded as the most well-known contributors of autoimmune diseases, including lupus, are miR-146a, miR-155 and miR-181a 111. According to the databases used for identifying target genes, the potential targets of miR-146a are TRAF-6 and IRAK-1, both key components of the TLR4 signaling pathway, which takes part in innate immunity against extracellular pathogens, such as bacteria.

Studies have shown that miR-146a acts to downregulate this innate immune response against bacterial pathogens 139, 140. MiR-155, on the other hand, plays a key role in both innate and adaptive immunity, by regulating the germinal center response of B cells and Ig class switching of plasma cells 141, 142. Lastly, miR-181a is reported to take part in the development of bone marrow-derived B-cells and CD8+ T-cells 143.

In addition to the three miRNAs mentioned above, in the field of lupus pathogenesis, miR-21, miR-125b, miR-148a have also been studied extensively 144. Both miR-21 and miR-148a level are found to be increased in CD4+ T-cells of SLE patients. They are reported to function as suppressors of DNA methyltransferase 1 (DNMT1) in T-cells, promoting hypomethylation and overexpression of autoimmune-associated genes like CD70 in T-cells 57. MiR-21 is also known to be overexpressed in B-cells and plasma of lupus patients. Research has found that overexpressed miR-21 in B-cells suppress the expression of phosphatase and tensin homolog (PTEN), thereby inducing the generation and activation of self-reactive B-cells 99. Meanwhile, the specific mechanism leading to increased levels of miR-21 in the plasma of lupus patients remains unclear. Scholars predict that it might be due to the secretion from circulatory CD4+ T-cells with upregulated miR-21, which are overly exposed to circulatory plasma in inflammatory conditions such as SLE 121. Furthermore, miR-146a and miR-155 levels in plasma, serum, and neutrophils were decreased in SLE patients, whereas their urine level was increased 35, 121, 125-128, 145, 146. MiR-181a levels in plasma of SLE patients were controversial among studies, but most have reported that its level was increased, in correlation with SLEDAI score 122, 131, 136-138.

Although dysregulated miRNA levels measured in B-cells, T-cells, plasma, and urine each have very different meanings, the type of specimen used to estimate miRNA levels tend to be overlooked in the past studies on miRNAs. Moreover, there is growing interest in the dysregulation of extracellular miRNAs clinically obtained from plasma, serum, and urine, partly due to their easy accessibilities. However, its physiological function, whether it participates in cell-to-cell communication or is just a byproduct of circulatory cell death, and its specific mechanism of selective secretion from circulatory cells still remains unclear 118, 119, 121, which calls for further studies on the functions of extracellular miRNA itself.

## Author Contributions

All authors made substantial contributions to all of the following: 1 conception and design of the study, data acquisition, or analysis and interpretation of data; 2 drafting or critical revision of the article for intellectual conduct; and 3 final approval of version to be submitted.

## Figures and Tables

**Table 1:** MiRNA profiles of systemic lupus erythematosus.

Sample type	miRNAs	
	Upregulated	Downregulated
**MiRNAs involved in SLE pathogenesis (SLE vs. HC, active SLE vs. inactive SLE, LNN vs. HC)**		
Renal tissue	miR-198 (147) miR-423-5p, miR-663a (148)	
Peripheral blood leukocytes		miR-146a (126)
PBMC	miR-629, miR-525-5p, miR-516a-3p (149) miR-125, miR-1273h-5p (150) miR-16, miR-21, miR-155 (151) miR-155 (152) miR-199-3p (153) miR-27a-5p (154) miR-29b (155) miR-873 (156)	miR-125a (157) miR-125b (158) miR-141 (151) miR-146a (159) miR-155, miR-17, miR-181b (160) miR-155 (161) miR-17-5p (162) miR-181a-3p, miR-409-5p, miR-410, miR-485-3p, miR-543, miR-654-3p (163) miR-17-5p (164) miR-155 (165)
T-cell	miR-126, miR-451, miR-181b (163) miR-126 (61) miR-224, miR-143 (65) miR-224 (53) miR-148a, miR-21 (57) miR-21 (59) miR-21-5p (58) miR-29b (60) miR-326 (69) miR-449b, miR-524-5p (54)	miR-125a-5p (62) miR-142-3p, miR-505, miR-324-5p (67) miR-142-3p (61) miR-142-3p, miR-142-5p (63) miR-145 (53) miR-31 (55) miR-410 (56) miR-633 (66) miR-98 (64)
B-cell	miR-29b, miR-494, miR-9 (166) miR-10a, miR-345 (65) miR-326 (102) miR-152-3p (101) miR-21, miR-22, miR-7 (99) miR-30a (100)	miR-29c, miR-181c, miR-223, miR-324-5p, miR-328, miR-362-3p, miR-744, let-7d, miR-7e, miR-26a, miR-335, miR-532, miR-579, miR-629 (166) miR-15b (103) miR-1246 (104)
Monocyte	miR-146a, miR-155 (35)	miR-124a, miR-125a (35) miR-19b, miR-20a (167) miR-302d (168)
NK cell	miR-27a-5p (154)	
Neutrophil		miR-124a, miR-125a, miR-222, miR-125b, miR-146a, miR-155 (35)
Extracellular sample (serum, plasma, urine)	miR-142-3p, miR-181a (131) miR-551b, miR-448 (169) miR-126, miR-16, miR-21, miR-223, miR-451 (121) miR-146a (128) miR-146a, miR-155 (127) miR-146a (125) miR-181a (136) miR-181a, miR-196a, miR-21 (122) miR-21 (124) miR-21 (123) miR-223-3p, miR-30e-5p, miR-92a-3p (170) miR-371-5p, miR-5100, miR-4642, miR-548a-5p, miR-518b, miR-4762-5p, miR-767-3p, miR-4708-3p (171)	miR-103, miR-150, miR-20a, miR-223, miR-27a, miR-15b, miR-16, miR-181a, miR-19b, miR-22, miR-23a, miR-25, miR-92a, miR-93 (138) miR-106a, miR-17, miR-20a, miR-203, miR-92a (131) miR-151a-3p, miR-148b-3p, miR-106b-3p, miR-28-5p (172) miR-124 (169) miR-125a-3p, miR-155, miR-146a (121) miR-126 (173) miR-200a, miR-200b, miR-200c, miR-429, miR-205, miR-192, miR-141 (174) miR-146a, miR-155 (125) miR-146a (146) miR-150-5p, miR-21-5p, miR-221-3p (175) miR-203a (136) miR-31 (123) miR-148b-3p, miR-146a-5p, miR-223-3p, miR-1246, miR-21-5p (171)
**MiRNAs in involved in LN pathogenesis (LN vs. HC, active LN vs. inactive LN, LN vs. LNN)**		
Renal tissue	let-7a, let-7e, let-7i, let-7g (176) miR-130b (177) miR-198, miR-146a, miR-638, miR-198 (178) miR-150 (179) miR-146a (180) miR-422a, miR-21 (181) miR-423-5p, miR-663a (148)	miR-130b (182) miR-146a (183) miR-638 (178) miR-23b (180) miR-26a, miR-30b, miR-4286 (184) miR-371-5p (185) miR-26a, miR-133 (181)
PBMC	miR-21, miR-155, miR-16, miR-21 (151)	miR-141 (151) miR-146a (186) miR-146a (159) miR-5571-5p, miR-766-3p (187)
T-cell	miR-126, miR-451, miR-181b (67)	miR-142-3p, miR-505, miR-324-5p (67)
B-cell	miR-145 (166)	miR-29c, miR-345, miR-21, miR-18b, miR-365 (134)
Extracellular sample (serum, plasma, urine)	miR-125a-5p, miR-146a-5p, miR-155-5p (188) miR-204-5p, miR-30c-5p (189) miR-130b-3p (190) miR-148a-3p (191) miR-150, miR-21 (192) miR-21-5p, miR-423-3p (193) miR-182-5p (194)* miR-3135b, miR-654-5p, miR-146a-5p (195)	let-7a, miR-21 (196) miR-3201, miR-1273e (189) miR-130b-3p (190) miR-200b-5p, miR-141-5p, miR-200c-5p (197) miR-410, miR-29c (192) miR-26a, miR-30b (184) miR-29c (198)

Abbreviations: RNA, ribonucleic acid; SLE, systemic lupus erythematosus; HC, healthy control; LNN, lupus nephritis-negative; PBMC, peripheral blood mononuclear cells; NK cell, natural killer cell. *measured in a whole blood sample

**Table 2:**
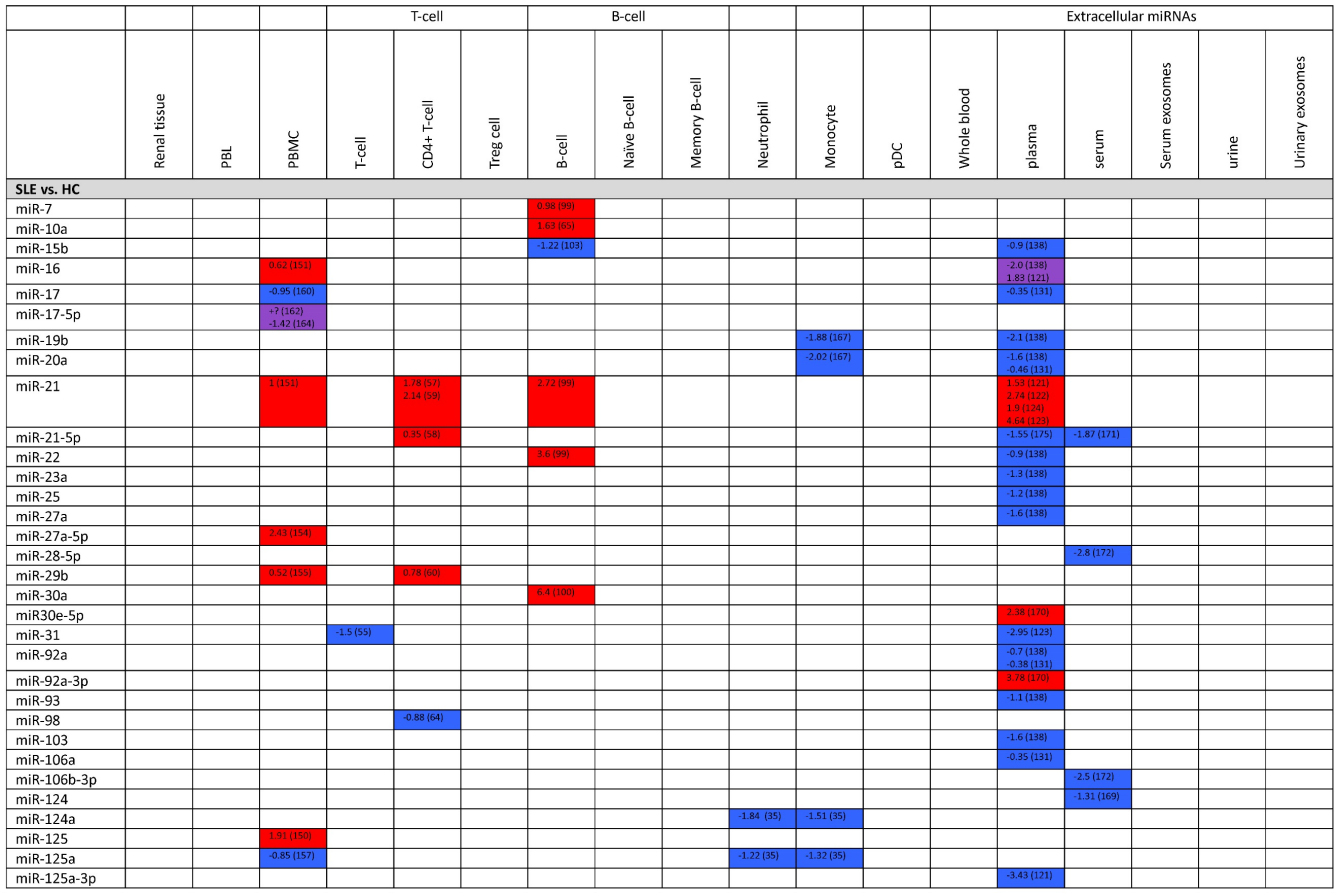

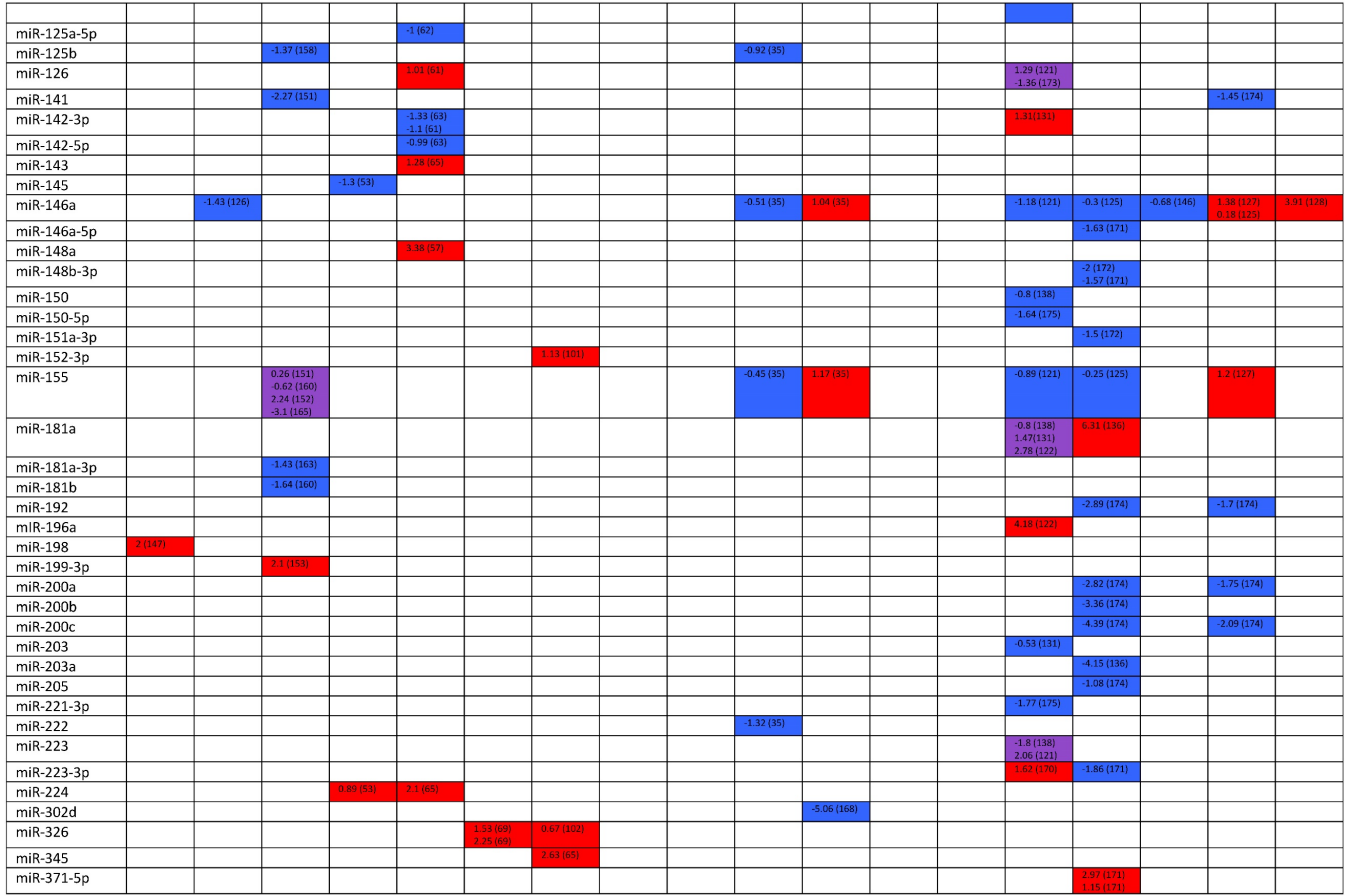

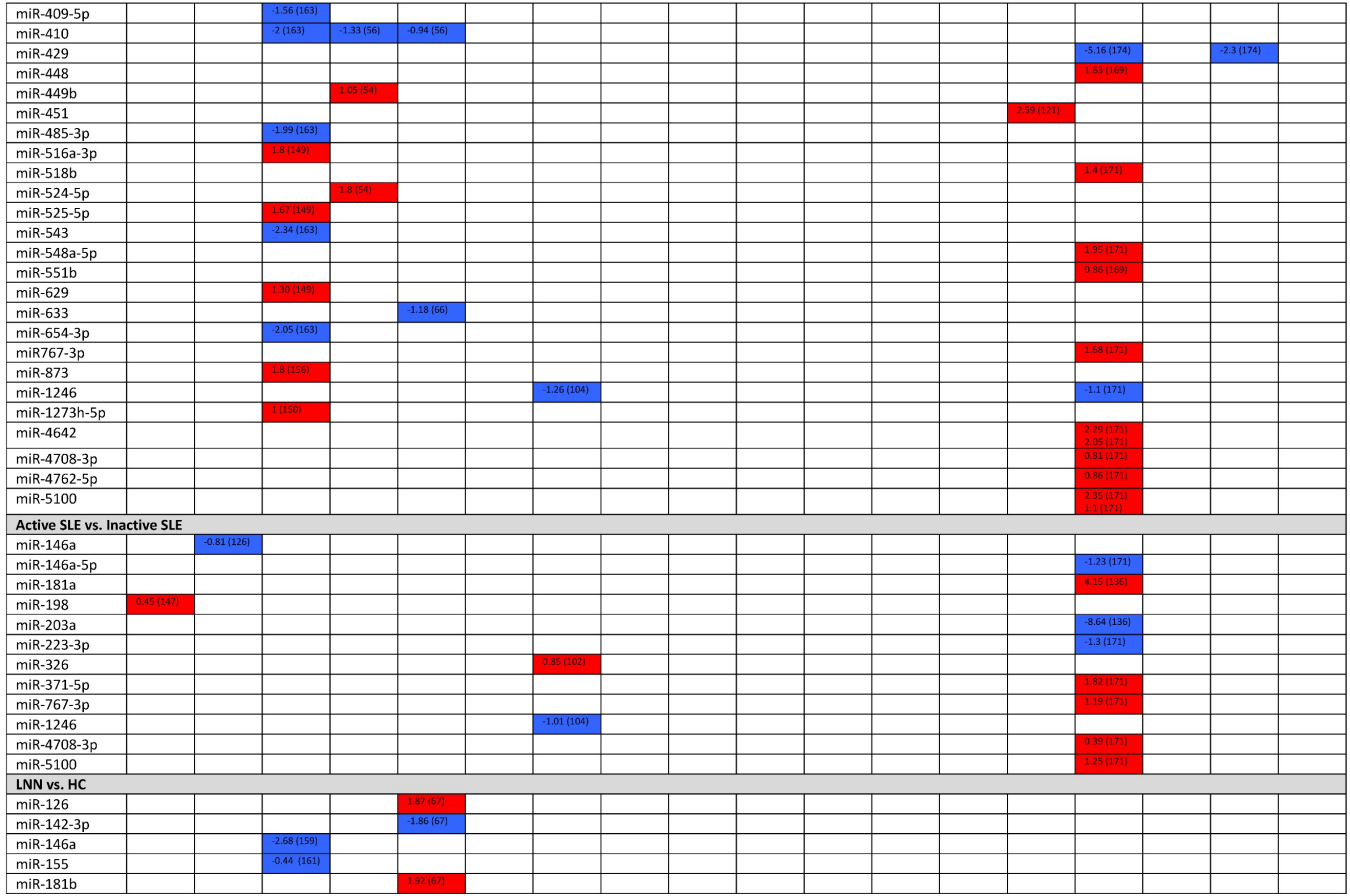

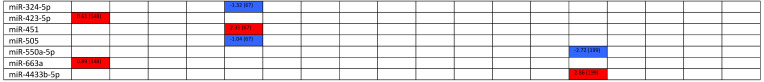
Expression levels of dysregulated miRNAs in SLE patients (expressed in log2FC values).

Abbreviations: FC, fold change; SLE, systemic lupus erythematosus; HC, healthy control; LNN, lupus nephritis-negative; PBL, peripheral blood leukocytes; PBMC, peripheral blood mononuclear cells; pDC, plasmacytoid dendritic cells.

**Table 3:**
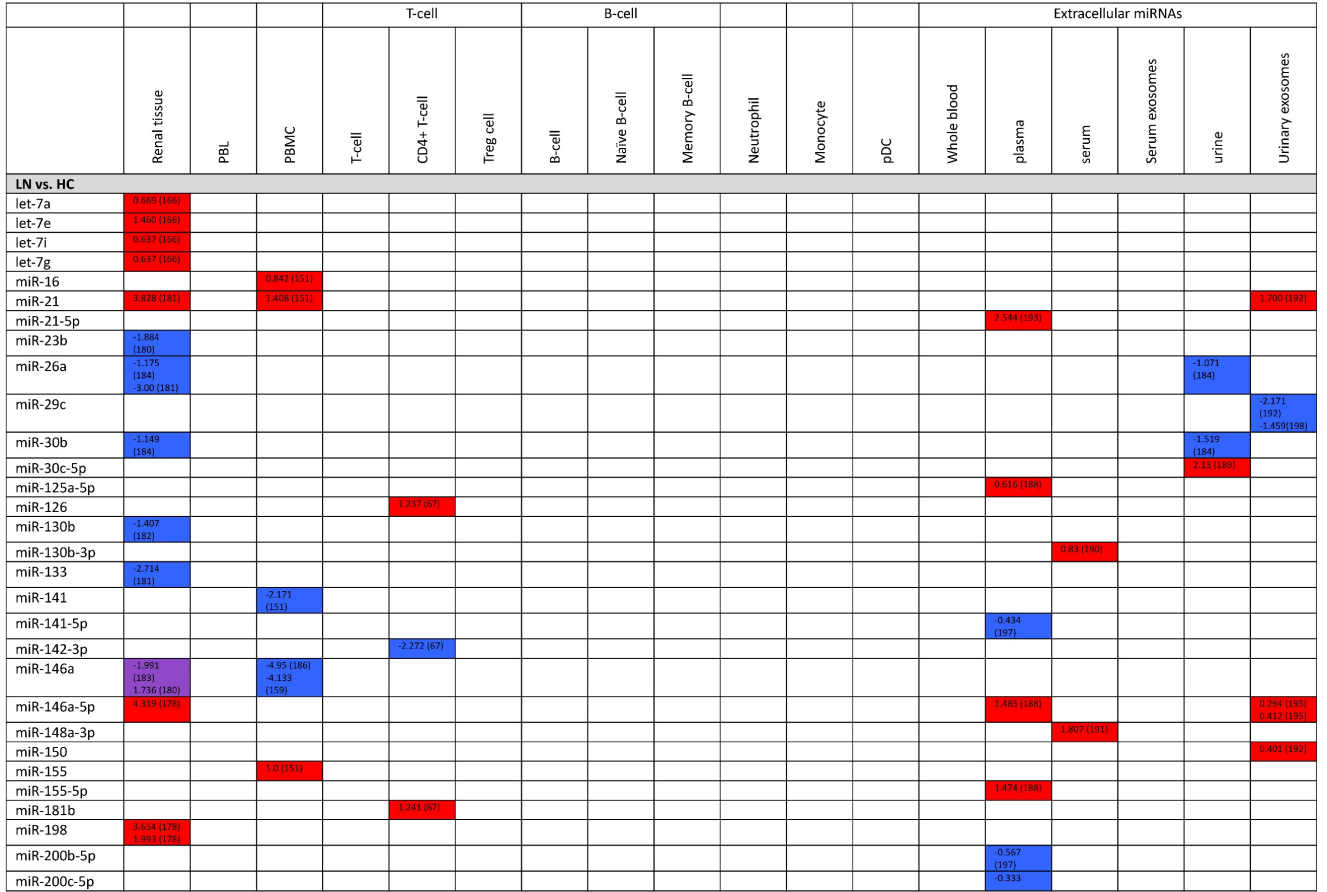

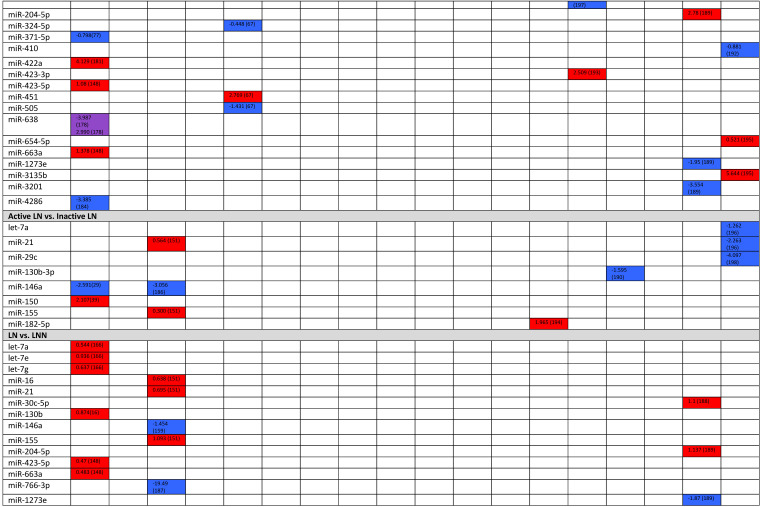

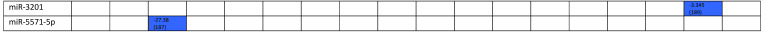
Expression levels of dysregulated miRNAs in LN patients (expressed in log2FC values).

Abbreviations: FC, fold change; LN, lupus nephritis; HC, healthy control; LNN, lupus nephritis-negative; PBL, peripheral blood leukocytes; PBMC, peripheral blood mononuclear cells; pDC, plasmacytoid dendritic cells.
